# A plant protein farnesylation system in prokaryotic cells reveals Arabidopsis AtJ3 produced and farnesylated in *E. coli* maintains its function of protecting proteins from heat inactivation

**DOI:** 10.1186/s13007-023-01087-x

**Published:** 2023-10-26

**Authors:** Jia-Rong Wu, Rida Zohra, Ngoc Kieu Thi Duong, Ching-Hui Yeh, Chung-An Lu, Shaw-Jye Wu

**Affiliations:** Department of Life Sciences, National Central University, 300 Jhong-Da Road, Jhong-Li District, Taoyuan, 32001 Taiwan

**Keywords:** Arabidopsis, AtJ3, HSP40, OsDjA4, Post-translational modification, Protein farnesylation, Protein farnesyltransferase, Heat stress, Thermotolerance

## Abstract

**Background:**

Protein farnesylation involves the addition of a 15-carbon polyunsaturated farnesyl group to proteins whose C-terminus ends with a CaaX motif. This post-translational protein modification is catalyzed by a heterodimeric protein, i.e., farnesyltransferase (PFT), which is composed of an α and a β subunit. Protein farnesylation in plants is of great interest because of its important roles in the regulation of plant development, responses to environmental stresses, and defense against pathogens. The methods traditionally used to verify whether a protein is farnesylated often require a specific antibody and involve isotope labeling, a tedious and time-consuming process that poses hazardous risks.

**Results:**

Since protein farnesylation does not occur in prokaryotic cells, we co-expressed a known PFT substrate (i.e., AtJ3) and both the α and β subunits of Arabidopsis PFT in *E. coli* in this study. Farnesylation of AtJ3 was detected using electrophoretic mobility using SDS-PAGE and confirmed using mass spectrometry. AtJ3 is a member of the heat shock protein 40 family and interacts with Arabidopsis HSP70 to protect plant proteins from heat-stress-induced denaturation. A luciferase-based protein denaturation assay demonstrated that farnesylated AtJ3 isolated from *E. coli* maintained this ability. Interestingly, farnesylated AtJ3 interacted with *E. coli* HSP70 as well and enhanced the thermotolerance of *E. coli*. Meanwhile, AtFP3, another known PFT substrate, was farnesylated when co-expressed with AtPFTα and AtPFTβ in *E. coli*. Moreover, using the same strategy to co-express rice PFT α and β subunit and a potential PFT target, it was confirmed that OsDjA4, a homolog of AtJ3, was farnesylated.

**Conclusion:**

We developed a protein farnesylation system for *E. coli* and demonstrated its applicability and practicality in producing functional farnesylated proteins from both mono- and dicotyledonous plants.

**Supplementary Information:**

The online version contains supplementary material available at 10.1186/s13007-023-01087-x.

## Background

Post-translational modification (PTM) is a chemical process that occurs after protein translation, where functional groups are covalently attached to proteins or proteolysis of proteins takes place; this occurs only in eukaryotes and not in prokaryotes [1, 2]. PTM can affect the structure, integrity, charge state, and hydrophobicity of proteins [3–8]. PTM is crucial in modulating specific protein functions in plant cells related to developmental and environmental cues, expanding proteome diversity, and increasing the functionality of cell signaling [9–11].

Protein lipidation is a PTM that refers to the process of adding lipid molecules to a protein to facilitate membrane localization and interaction with other proteins [12]. Among various protein lipidation, protein farnesylation is the attachment of a 15-carbon farnesyl isoprenoid lipid to proteins containing a CaaX motif at their C-terminus, where the “C” is cysteine for thioester linkage of the farnesyl group, “a” is an aliphatic amino acid, and “X” can be any amino acid; however, is usually alanine, serine, methionine, or glutamine [13]. The enzyme that catalyzes this modification reaction is a heterodimeric protein farnesyl transferase (PFT), which is composed of an α and a β subunit [13, 14]. Protein farnesylation has gained great attention in plant science since the isolation of the Arabidopsis *enhanced response to aba 1* (*era1*) mutant. This mutant was isolated for its hypersensitivity to stress hormone abscisic acid (ABA)-induced phenotypes, including seed dormancy, stomatal closure, and drought tolerance, and the phenotypes were caused by a lesion in the PFTβ coding gene (AT5G40280) [15]. Soon afterward, it was discovered that mutations in *ERA1* also influence floral meristem organization, increasing the number of organs in floral whorls and homeotic transformations of flowers [16–18]. Later, *era1* displayed enhanced susceptibility to bacterial and fungal infections [19]. More recently, an Arabidopsis *heat-intolerant 5* (*hit5*) mutant that could not survive prolonged heat stress treatment was isolated, and the mutated locus was identified to be in the PFT β coding gene [11]. These findings not only demonstrate the versatile roles of protein farnesylation in regulating plant growth and development but also indicate its participation in plant responses to both biotic and abiotic stresses. Because these traits derived from protein farnesylation are of agronomic interest, a better understanding of the regulatory network mediated by protein farnesylation could provide a strategy for enhancing stress tolerance in plants and improving crop yield. However, it is necessary to identify the candidate PFT substrates and verify whether they are farnesylated.

Using publicly available proteomic databases, candidate PFT targets can be easily identified. For example, approximately 120 potential PFT-targeting proteins whose C-terminal amino acid sequence ends with CaaX have been identified in Arabidopsis [20]. However, it is challenging to determine whether these potential PFT targets are farnesylated. Traditionally, this is achieved by feeding radioactive isotope-labeled farnesol, which is converted into farnesyl pyrophosphate (FPP), the donor molecule used in the process of protein farnesylation, to plants [21, 22]. Total proteins extracted from plants are then subjected to immunoprecipitation to isolate candidate proteins. Finally, farnesylation of candidate proteins is detected using autoradiography. This method is very time-consuming, necessitates the availability of a specific antibody, and poses hazardous risks during its execution [2]. Moreover, it has the drawback of being unable to produce large-scale protein preparations for crystallographic or biophysical investigations. Alternatively, various FPP analogs have been developed for immunofluorescent or fluorescent detection of farnesylated proteins [23–26]. Nonetheless, this strategy is useful for studying farnesylated proteomes and PFT inhibitors but not validating individual PFT targets. Anti-farnesyl antibodies that can detect specific farnesylated proteins have been raised [27–29]. However, these antibodies may cross-react with proteins modified by other lipids [30, 31]. In contrast, it has been reported that recombinant mouse PFTα, PFTβ, and PFT targeting liver kinase B1 (LKB1) genes can be expressed separately in *E. coli* and then purified for use in in vitro protein farnesylation reactions. This method requires exogenous supplementation of FPP but eliminates isotope labeling, and the farnesylation of LKB1 is confirmed using mass spectrometry (MS) [28]. Meanwhile, it has been demonstrated that human PFT targeting guanylate-binding protein 1 (hGBP1) can be farnesylated in vivo when its cDNA is co-transformed and co-expressed with human PFTα and PFTβ cDNAs in *E. coli*, showing that the farnesylated hGBP1 maintained its biological function [32]. These *E. coli* systems simplify the verification procedure and facilitate the characterization of potential PFT targets. Nevertheless, these systems have only been successfully applied to PFT and animal-origin substrates; whether they apply to those of plant origin is yet to be tested.

The Arabidopsis J3 protein (AtJ3; AT3G44110), a member of the heat shock protein 40 (HSP40) family and containing a CaaX motif (CAQQ) at its C-terminus, was previously verified as a PFT substrate using the isotope labeling method [33]. Later, it was shown that plants lacking farnesylated AtJ3 display similar heat-dependent phenotypes, such as *hit5/era1* [34]. To test the applicability of protein farnesylation with components from plants in *E. coli*, cDNAs encoding AtJ3, AtPFTα, and AtPFTβ were co-transformed into and expressed in *E. coli* in this study. Farnesylation of AtJ3 was easily detected using a mobility shift assay and confirmed using mass spectrometry. Then, cDNA encoding an AtJ3 homolog from *Oryza sativa*, OsDjA4 (Os03g44620), was introduced into and co-expressed with cDNAs encoding OsPFTα and OsPFTβ in *E. coli*. Farnesylated OsDjA4 was also detected and confirmed. Furthermore, the farnesylated protein produced in *E. coli* in this study contained an affinity tag; thus, pure and sufficient quantity of the target proteins can be generated for molecular study. In vitro analysis showed that farnesylated AtJ3 produced in *E. coli* maintained its ability to protect Arabidopsis proteins from heat-induced damage, as the farnesylated AtJ3 should readily bind to the exposed hydrophobic stretches of denatured proteins and facilitate their interaction with HSP70 [35]. Interestingly, *E. coli* containing farnesylated AtJ3 exhibited better thermotolerance than those containing unfarnesylated AtJ3, indicating that prokaryotic cells can benefit from farnesylation to tolerate heat stress. The mechanism underlying this phenomenon is discussed.

## Materials and methods

###  Cloning of cDNA

To clone *AtJ3* (AT3G44110), *AtFP3* (At5g63530), *AtPFTα* (AT3G59380), and *AtPFTβ* (AT5G40280), DNAse-treated RNA that had been isolated from Arabidopsis rosette leaves was reverse-transcribed using Moloney Murine Leukemia Virus HP Reverse Transcriptase (Epicentre Technologies, Madison, WI, USA) using an oligo(dT) primer to generate first-strand cDNA. *AtJ3* cDNA was amplified using the primers AtJ3-6xHis-BamHI-F and AtJ3-stop-XhoI-R, *AtFP3* using AtFP3-6xHis-BamHI-F and AtFP3-stop-EcoRI-R, *AtPFTα* using AtPFT-α-BamHI-F and-PstI-R, and *AtPFTβ* using AtPFT-β-6xHis-KpnI-F and AtPFT-β-PacI-R. The resulting amplicon of *AtJ3* was digested with *Bam*HI and *Xho*I and cloned into the bacterial expression vector pGEX-4T-1 downstream of a Lac operator and the GST sequence to generate pGEX-AtJ3. Therefore, the expression of *AtJ3* in this vector required IPTG induction, and the AtJ3 produced was tagged with GST. Similarly, the resulting amplicon of *AtFP3* was digested with *Bam*HI and *Eco*RI and cloned into the pGEX-4T-1 to generate pGEX-AtFP3. The resulting amplicon of *AtPFTα* was digested with *Bam*HI and *Pst*I, *AtPFTβ* with *Kpn*I and *Pac*I, and cloned into multiple cloning site-1 (MCS-1) and − 2 (MCS-2) of the bacterial expression vector pRSF-Duet1 to generate pRSF-Duet1-AtPFT. Both MCS-1 and MCS-2 are individually downstream of a Lac operator; therefore, their expression also requires IPTG induction. All recombinant proteins derived from this design were tagged with a 6xHis sequence which facilitated the detection of their expression in *E. coli.*

The strategy used to clone Arabidopsis *AtJ3, AtPFTα*, and *AtPFTβ* was employed to clone rice *OsDjA4* (Os03g44620), *OsPFTα* (Os09g33930), and *OsPFTβ* (Os01g53600). *OsDjA4* cDNA was amplified using primers OsDjA4-6xHis-SmaI-F and OsDjA4-stop-NotI-R, *OsPFTα* with OsPFTα-BamHI-F and OsPFTα-EcoRI-R, and OsPFTβ with OsPFTβ-6xHis-KpnI-F and OsPFTβ-XhoI-R The resulting amplicon of *OsDjA4* was digested with *Sma*I and *Not*I and cloned into the bacterial expression vector pGEX-4T-1 downstream of the Lac operator and GST sequence to generate pGEX-OsDjA4. The resulting amplicon of *OsPFTα* was digested with *Bam*HI and *Eco*RI, *OsPFTβ* with *Kpn*I and *Xho*I, and cloned into MCS-1 and MCS-2 of pRSF-Duet1, respectively, to produce pRSF-Duet1-OsPFT. Again, all the recombinant proteins derived from this design were tagged with a 6xHis sequence which facilitated the detection of their expression in *E. coli.* The primers used for cloning cDNAs are shown in Additional file 1: Table S1.

### Induction of recombinant J and PFT proteins

To test Arabidopsis protein farnesylation in *E. coli*, pGEX-AtJ3 and pRSF-Duet1-AtPFT were simultaneously co-transferred into *E. coli* Rosetta competent cells using a heat shock procedure at 42 °C for 90 s. The transformed cells were then plated onto solid LB media containing 50 µg/mL carbenicillin and 50 µg/mL kanamycin to select pGEX-AtJ3 and pRSF-Duet1-AtPFT, respectively. Viable colonies were further genotyped using PCR with specific primers to ensure the accurate selection of co-transformed cells. A successful transformant was grown in Luria Bertani (LB) medium containing appropriate antibiotics at 37 °C. Once the optical density (600 nm) of bacterial suspension reached 0.6–0.8, 100 mM isopropyl β-D-1-thiogalactopyranoside (IPTG) was added, and the cultures were incubated at 37 °C for 22 h to induce the proteins. An identical procedure was applied to induce *E. coli* cells co-transformed with pGEX-OsDjA4 and pRSF-Duet1-OsPFT to test rice protein farnesylation.

### Identification of the production of recombinant J and PFT proteins

After induction with IPTG, cells from 3 mL of suspension culture were harvested by centrifugation. Total proteins from these cells were extracted using 2x sample buffer (20% glycerol, 6% SDS, 0.22 M Tris [pH 6.8], 0.01% bromophenol blue, and 10% β-mercaptoethanol), heated at 95 °C for 5 min to denature the proteins, and then centrifuged at 12,500 rpm for 22 min at 4 °C. Subsequently, 20 µL supernatant of total proteins was loaded onto a 12.5% sodium dodecyl sulfate-polyacrylamide gel electrophoresis (SDS-PAGE) gel to separate the proteins and assess the induction of J and PFT proteins. Afterward, the gel was stained with 0.1% Coomassie brilliant blue (CBB) R-250, 50% methanol, and 10% glacial acetic acid to visualize the protein bands. The proteins from a second gel were transferred onto a PVDF membrane using a cold transfer buffer (25 mM Tris base, 200 mM glycine, and 20% methanol) at 100 V for 70 min for immunoblotting. The membrane was rinsed 3 times for a minimum of 10 min each with TTBS buffer (20 mM Tris base, 500 mM NaCl, 0.05% TritonX-100) and TBS buffer (20 mM Tris base and 500 mM NaCl) to remove any residual substances. After rinsing, the membrane was incubated overnight at 4 ℃ on a shaker with a solution containing anti-6xHis primary antibody (Proteintech, Rosemont, IL, USA). The following day, the membrane was washed with TTBS and TBS and incubated with a horseradish peroxidase (HRP)-linked secondary antibody (goat anti-mouse #115-035-003, Jackson Immuno Research, West Grove, PA, USA) with shaking for 3–4 h at 4 °C, followed by washing with TTBS and TBS. Finally, HRP activity was detected using western lightning chemiluminescence reagent (Perkin Elmer, San Francisco, CA, USA) and visualized using a luminescent image analyzer (ImageQuantTM LAS-4000, FujiFilm, Valhalla, NY, USA).

### Purification of J protein from single and co-transformed cells

To purify GST-tagged AtJ3 from *E. coli* transformed with either pGEX-AtJ3 or co-transformed with pGEX-AtJ3 and pRSF-Duet1-AtPFT, 500 mL liquid culture induced with IPTG was centrifuged at 6000 rpm for 10 min. The resulting pellets were resuspended in 20 mL of lysis buffer containing 140 mM NaCl, 2.7 mM KCl, 10 mM Na_2_HPO_4_, 1.8 mM KH_2_PO_4_, 10% glycerol, 0.1% Triton X-100, 1 mM DTT, and 0.1 mM PMSF. The bacterial cells were disrupted using an ultrasonic processor and centrifuged at 10,000 rpm for 30 min at 4 °C to separate the supernatant containing the proteins. Glutathione Sepharose 4 B beads (Cytiva, Marlborough, MA, USA) were used to isolate GST-tagged AtJ3 according to the manufacturer’s instructions. The GST tag was removed from AtJ3 by digestion with thrombin, resulting in 6xHis-AtJ3. This procedure was also used to isolate GST-tagged OsDjA4 from *E. coli* transformed with pGEX-OsDjA4, or co-transformed with pGEX-OsDjA4 and pRSF-Duet1-OsPFT to obtain 6xHis-OsDjA4.

### Verification of J protein farnesylation in *E. coli*

AtJ3 purified from *E. coli* transformed with pGEX-AtJ3 or co-transformed with pGEX-AtJ3 and pRSF-Duet1-AtPFT, and OsDjA4 purified from *E. coli* transformed with pGEX-OsDjA4 or co-transformed with pGEX-OsDjA4, and pRSF-Duet1-OsPFT were characterized using mass spectrometry. The protein bands corresponding to AtJ3 and OsDjA4 after SDS-PAGE were de-stained and then reduced with 10 mM dithiothreitol (DTT) at 60 °C for 45 min, followed by cysteine-blocking with 55 mM iodoacetamide (IAM) at 25 °C for 30 min. The samples were digested with trypsin at 37 °C for 16 h. The resulting peptides were extracted from the gel, dried using a vacuum centrifuge, and reconstituted in 0.1% formic acid before analysis.

The LC-MS/MS analysis was conducted by diluting the digested peptides in HPLC buffer A (0.1% formic acid) and loading them onto a reverse-phase column. The desalted peptides were separated using a multistep gradient of HPLC buffer B (99.9% acetonitrile/0.1% formic acid) for 70 min at a flow rate of 0.3 µL/min. The LC apparatus was coupled with a 2D linear ion trap mass spectrometer operated using Xcalibur 2.2 software. Full-scan MS over a range of 400–2,000 Da and a resolution of 120,000 at m/z 400 was performed using an Orbitrap ion trap mass analyzer. Protein and post-translational modification site identification was performed using the Proteome Discoverer software (version 2.2, Thermo Fisher Scientific). MS/MS spectra were searched against a customized database using the Mascot search engine (Matrix Science, London, UK). A mass tolerance of 10 ppm was permitted for intact peptide masses and CID fragment ions (0.5 Da), with an allowance for up to two missed cleavages made from the Lys-C digestion. Variable modifications included acetyl (protein N-terminal), oxidized (methionine), carbamidomethyl (cysteine), and farnesyl (cysteine). Peptide-spectrum matches (PSMs) were filtered based on high confidence and a Mascot search engine rank 1 for peptide identification to ensure an overall false discovery rate below 0.01. Proteins with a single peptide hit were removed to increase confidence in the identified proteins and PTM sites.

### Generation of Arabidopsis *j3* mutant harboring *Pro*_*LeHsp23.8*_ driven firefly luciferase gene

The *Pro*_*LeHsp23.8*_-Luciferase transgenic line [36] was crossed with the Arabidopsis *j3* mutant line (SALK141625) to generate an AtJ3-null mutant line containing the thermolabile firefly luciferase gene (Fluc) under the control of a highly heat-inducible *LeHsp23.8* (*atj3/Fluc*) [36, 37]. For testing heat-induced luciferase gene expression and enzyme activity in *anj3/Fluc* plants, the luciferase assay reagent (Promega E1500, Madison, WI, USA) was directly applied onto leaves of 9-day-old seedlings which had been incubated at 37 °C for 2 h and the luminescent image was captured using an image analyzer (ImageQuant LAS-4000, FujiFilm, Valhalla, NY, USA). To analyze the chaperone ability of AtJ3 produced in *E. coli*, 9-day-old heat-treated *atj3/Fluc* seedlings were homogenized in liquid nitrogen and added to luciferase cell lysis buffer (Promega E1531, Madison, WI, USA). About 30 µL of supernatant was then mixed with farnesylated and unfarnesylated AtJ3 proteins, which were produced and purified from *E. coli*. These mixtures were incubated at 44 °C for varying durations (0, 3, and 5 min). Subsequently, 50 µL of the luciferase assay reagent (Promega E1500, Madison, WI, USA) was added to the mixtures, and the luciferase activity was measured by the luminescence intensity using a Thermo Fluoroskan Ascent FL luminometer (Thermo Fisher, Waltham, MA USA).

### Thermotolerance assay of *E. coli* cells

*E. coli* cells containing pRSF-Duet1-AtPFT, pGEX-AtJ3, or pGEX-AtJ3 were generated. These cells were cultured individually in 2 mL of LB medium for 16 h and then transferred to 20 mL medium containing antibiotics to achieve an OD 600 of 0.6–0.8. IPTG was added to induce protein expression for 4 h. Subsequently, 1 mL of cells from each sample was subjected to heat stress by incubating them in a water bath set at 52 °C for 5 min under shaking at 150 rpm. Following heat stress, 100 µL of cells were taken from each sample and grown on LB plates at 37 °C for 16 h. The colonies obtained were counted to determine the farnesylated and unfarnesylated AtJ3 survival ratios.

### BiFC vector construction

In the execution of the BiFC assay, the AtJ3, DnaJ, and Dnak genes were transferred from their individual entry clones into the pBiFCt-2 in1-NN Gateway destination vector, following the methodology described in Grefen and Blatt, 2012 [38]. Subsequently, the nYFP-AtJ3 was amplified using the primers BiFCt-2in1-NN-(531–551)-BamHI-F and AtJ3-stop-EcoRI-R, nYFP-DnaJ using BiFCt-2in1-NN-(531–551)-BamHI-F and DnaJ-stop-EcoRI-R, and cYFP-DnaK using the BiFCt-2in1-NN-(3906–3926)-NdeI-F and DnaK-stop-XhoI-R. The resulting amplicons of nYFP-AtJ3 and nYFP-DnaJ were digested with *Bam*HI and *Eco*RI, the cYFP-DnaK with *Nde*I and *Xho*I. The digested fragments were cloned into the pRSF-Duet1 to generate pRSF-Duet1-nYFP-AtJ3-cYFP-DnaK and pRSF-Duet1-nYFP-DnaJ-cYFP-DnaK for BiFC assay in *E.coli* cells. The primers used for constructing BiFC vectors are shown in Additional file 1: Table S1. The *E. coli* cells were transformed with a construct containing nYFP-tagged AtJ3 (nYFP-AtJ3) and cYFP-tagged DnaK (cYFP-DnaK) to assess the interaction between AtJ3 and *E. coli* HSP70 (DnaK). The fluorescence of the YFP signal was observed and captured using an Olympus IX71 fluorescence microscope (Center Valley, PA, USA).

## Results

### AtPFTα, AtPFTβ, and AtJ3 can be produced concomitantly in *E. coli*

**Fig. 1:**
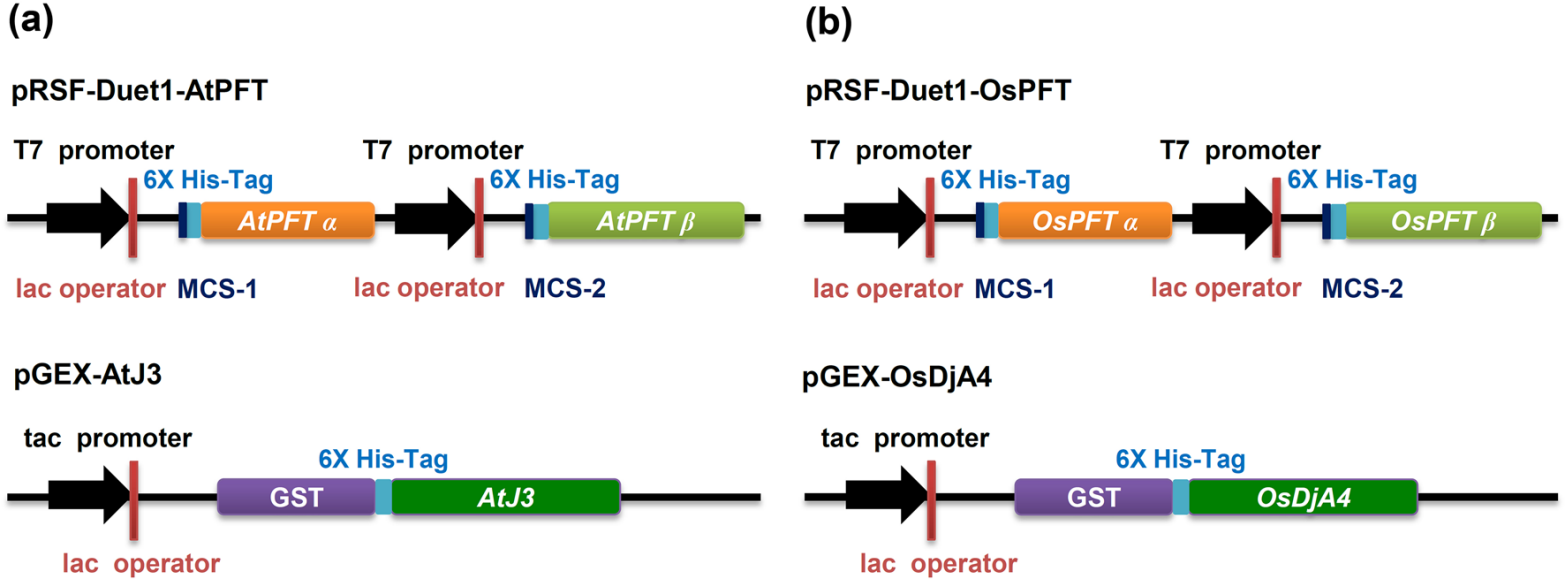
Schematic diagram illustrating the structure of vectors for plant protein farnesylation system in *E. coli*. **a** Arabidopsis *AtPFTα* and *AtPFTβ* cDNAs were cloned into multiple cloning sites 1 (MCS-1) and 2 (MCS-2) of pRSF-Duet1, respectively, to generate pRSF-Duet1-AtPFT. Both *AtPFTα* and *AtPFTβ* cDNA were fused with a 6x His-tag sequence downstream of a lac operator. Arabidopsis *AtJ3* cDNA was cloned into expression vector pGEX-4T-1 to generate pGEX-AtJ3. In pGEX-AtJ3, *AtJ3* was fused with both a GST and a 6x His-tag sequence for affinity purification and immunoblot identification. *AtJ3* cDNA was also downstream of a lac operator for ITPG induction. **b** Rice *OsPFTα* and *OsPFTβ* cDNA were cloned into MCS-1 and MCS-2 of pRSF-Duet1 to generate pRSF-Duet1-OsPFT; rice *OsDjA4* cDNA was cloned into pGEX-4T-1 to generate pGEX-OsDjA4.

**Fig. 2:**
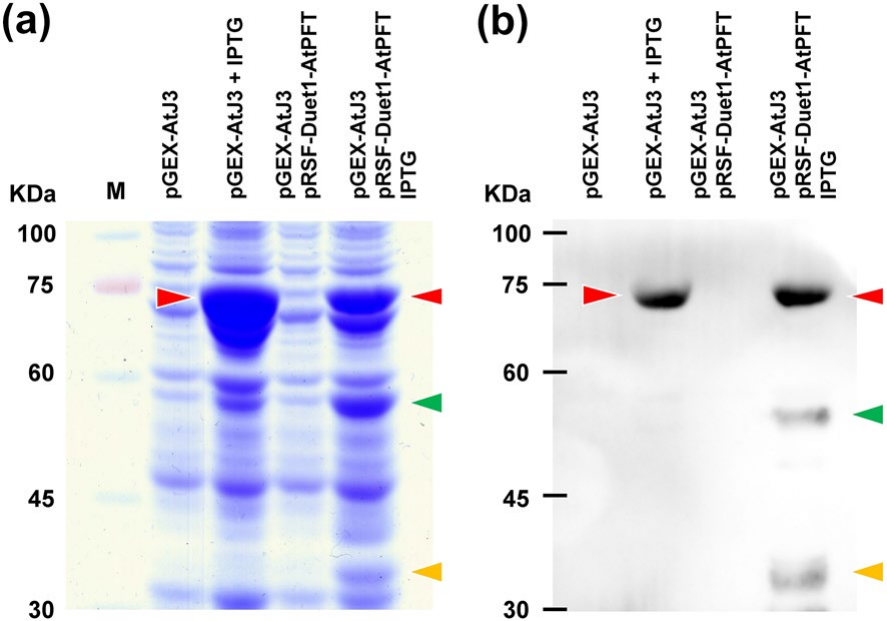
Co-expression of AtPFTα, AtPFTβ, and AtJ3 in *E. coli*. **a** Coomassie blue staining of a gel with proteins extracted from *E. coli* cells containing either pGEX-AtJ3 alone or both pGEX-AtJ3 and pRSF-Duet1-AtPFT before or after IPTG induction. **b** The protein samples in (a) were analyzed using western blot analysis with anti-6xHis-tag antibody. The molecular size of GST-6xHis-AtJ3 (72.67 kDa, red arrowhead), 6xHis-AtPFTβ (54.99 kDa, green arrowhead), and 6xHis-AtPFTα (38.78 kDa, orange arrowhead) are indicated

To test protein farnesylation catalyzed by Arabidopsis PFT in *E. coli*, *AtPFTα* and *AtPFTβ* cDNAs were inserted into the prokaryotic expression vector pRSF-Duet1 (pRSF-Duet1-AtPFT) and *AtJ3* into pGEX-4T-1 (pGEX-AtJ3). pRSF-Duet1 contains the RSF1013 origin of replication and can therefore be propagated in *E. coli* cells containing pGEX-4T-1 with a compatible ColE1 origin. *AtPFTα* and *AtPFTβ* cDNAs were designed to contain a 6x His-tag sequence. In contrast, *AtJ3* contains a 6x His-tag and a GST sequence. Their expressions in *E. coli* were regulated via IPTG induction (Fig. 1). Total proteins from *E. coli* cells co-transformed with pRSF-Duet1-AtPFT and pGEX-AtJ3, with or without IPTG induction, were extracted and separated using SDS-PAGE, followed by Coomassie blue staining. Proteins from IPTG induction cells displayed three distinct bands that were absent in those from non-IPTG-treated cells. The molecular weight of these three bands coincided with those of recombinant AtPFTα, AtPFTβ, and AtJ3 (Fig. 2a, Additional file 1: Fig. S1). To confirm these three protein bands representing AtPFTα, AtPFTβ, and AtJ3, western blot analysis was performed. These three protein bands were recognized by anti-His-tag antibodies (Fig. 2b, Additional file 1: Fig. S1), demonstrating that AtPFTα, AtPFTβ, and AtJ3 were produced concomitantly in *E. coli* cells.

### AtPFT can farnesylate AtJ3 and AtFP3 in *E. coli*

**Fig. 3:**
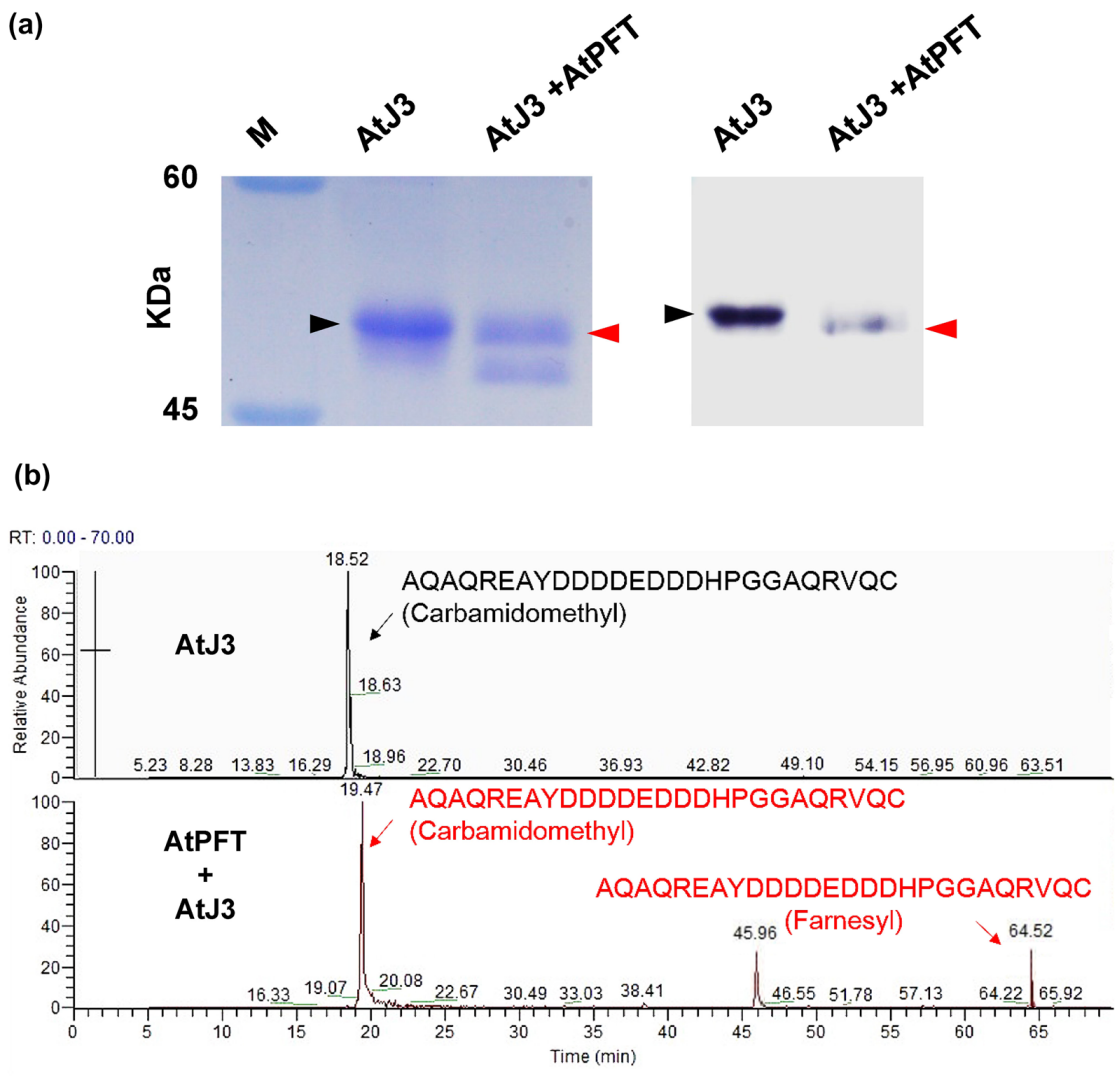
AtJ3 was farnesylated in AtPFT-producing *E. coli* cells. **a** AtJ3 produced in *E. coli* cells harboring either pGEX-AtJ3 alone or both pGEX-AtJ3 and pRSF-Duet1-AtPFT was purified using Glutathione Sepharose 4B beads and run in duplicate, one for Coomassie blue staining (left panel) and one for western blot detection (right panel), using SDS-PAGE. The AtJ3 purified from pGEX-AtJ3 and pRSF-Duet1-AtPFT co-transformed *E. coli* cells (AtPFT + AtJ3, red arrowhead) exhibited faster electrophoretic mobility than that from cells harboring pGEX-AtJ3 alone (AtJ3, black arrowhead), indicating AtJ3 from co-transformed cells was farnesylated. **b** The extracted ion chromatograms (EIC) of the C-terminal peptide of AtJ3 with and without farnesylation. The target peptide (AQAQREAYDDDDEDDDHPGGAQRVQC) produced from the cells containing pGEX-AtJ3 alone (upper panel) showed only a non-farnesylated form with 741.0565 (4+) m/z at 18.52 min, but the target peptide produced from the cells containing pRSF-Duet1-AtPFT and pGEX-AtJ3 (lower panel) showed both non-farnesylated form with 741.0565 (4+) m/z at 19.47 min and farnesylated form with 1036.7902 (3+) m/z at 64.52 min, respectively

To analyze whether AtPFT can farnesylate AtJ3 in *E. coli*, total proteins from cells harboring both pRSF-Duet1-AtPFT and pGEX-AtJ3 and pGEX-AtJ3 were extracted separately, followed by affinity purification using GST pull-down experiments to isolate AtJ3. Purified AtJ3s were subjected to SDS-PAGE and western blotting analyses. The results showed that AtJ3 purified from cells harboring pRSF-Duet1-AtPFT and pGEX-AtJ3 had faster electrophoretic mobility than cells harboring pGEX-AtJ3 (Fig. 3a, Additional file 1: Fig. S2). This is consistent with previous studies showing that farnesylated proteins run faster than their non-farnesylated counterparts on SDS-PAGE due to improved micelle formation of the farnesyl group with SDS [33, 39]. Purified AtJ3 from both cell types was subjected to MS analysis according to lipidated protein fragment-ion characteristics. The results showed that there was a farnesyl moiety attached to the cysteine residue of the CaaX box in AtJ3 purified from *E. coli* cells harboring pRSF-Duet1-AtPFT, but not from cells lacking pRSF-Duet1-AtPFT (Fig. 3b). These results concluded that Arabidopsis PFT α and β subunits could be produced and maintained their molecular function to recognize the CaaX box and execute protein farnesylation in *E. coli*. To further strengthen the feasibility of this *E. coli*-based plant protein farnesylation system, the *AtJ3* cDNA sequence in pGEX-AtJ3 was substituted by Arabidopsis *AtFP3* to make pGEX-AtFP3. AtFP3 is another known AtPFT target [43]. Similarly, total proteins from cells harboring both pRSF-Duet1-AtPFT and pGEX-AtFP3 and pGEX-AtJ3 were extracted separately, followed by affinity purification using GST pull-down experiments to isolate AtFP3. Purified AtFP3s were then subjected to SDS-PAGE and western blotting analyses. The results showed that AtFP3 purified from cells harboring pRSF-Duet1-AtPFT and pGEX-AtJ3 had faster electrophoretic mobility than cells harboring pGEX-AtFP3, indicating that AtFP3 was farnesylated in *E. coli* expressing AtPFT (Additional file 1: Fig. S3).

### OsPFT can farnesylate OsDjA4 in *E. coli*

**Fig. 4:**
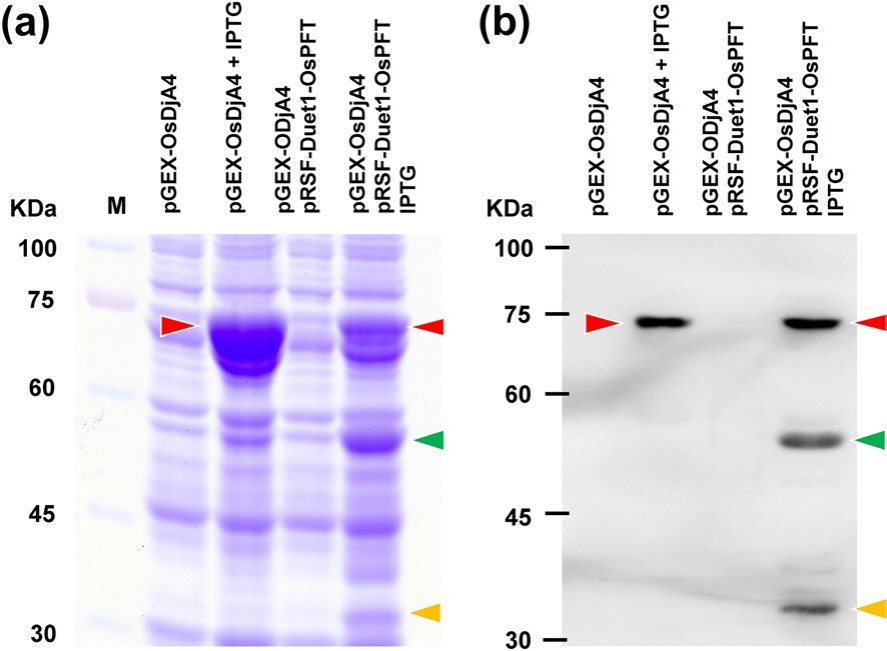
Co-expression of OsPFTα, OsPFTβ, and OsDjA4 in *E. coli*. **a** Coomassie blue staining of a gel with proteins extracted from *E. coli* cells harboring either pGEX-OsDjA4 alone or both pGEX-OsDjA4 and pRSF-Duet1-OsPFT, before or after IPTG induction. **b** The protein samples in (a) were analyzed using western blot analysis with an anti-6xHis-tag antibody. The molecular size of GST-6xHis-OsDjA4 (73.11 kDa, red arrowhead), 6xHis-OsPFTβ (53.43 kDa, green arrowhead), and 6xHis-AtPFTα (38.2 kDa, orange arrowhead) are indicated

**Fig. 5:**
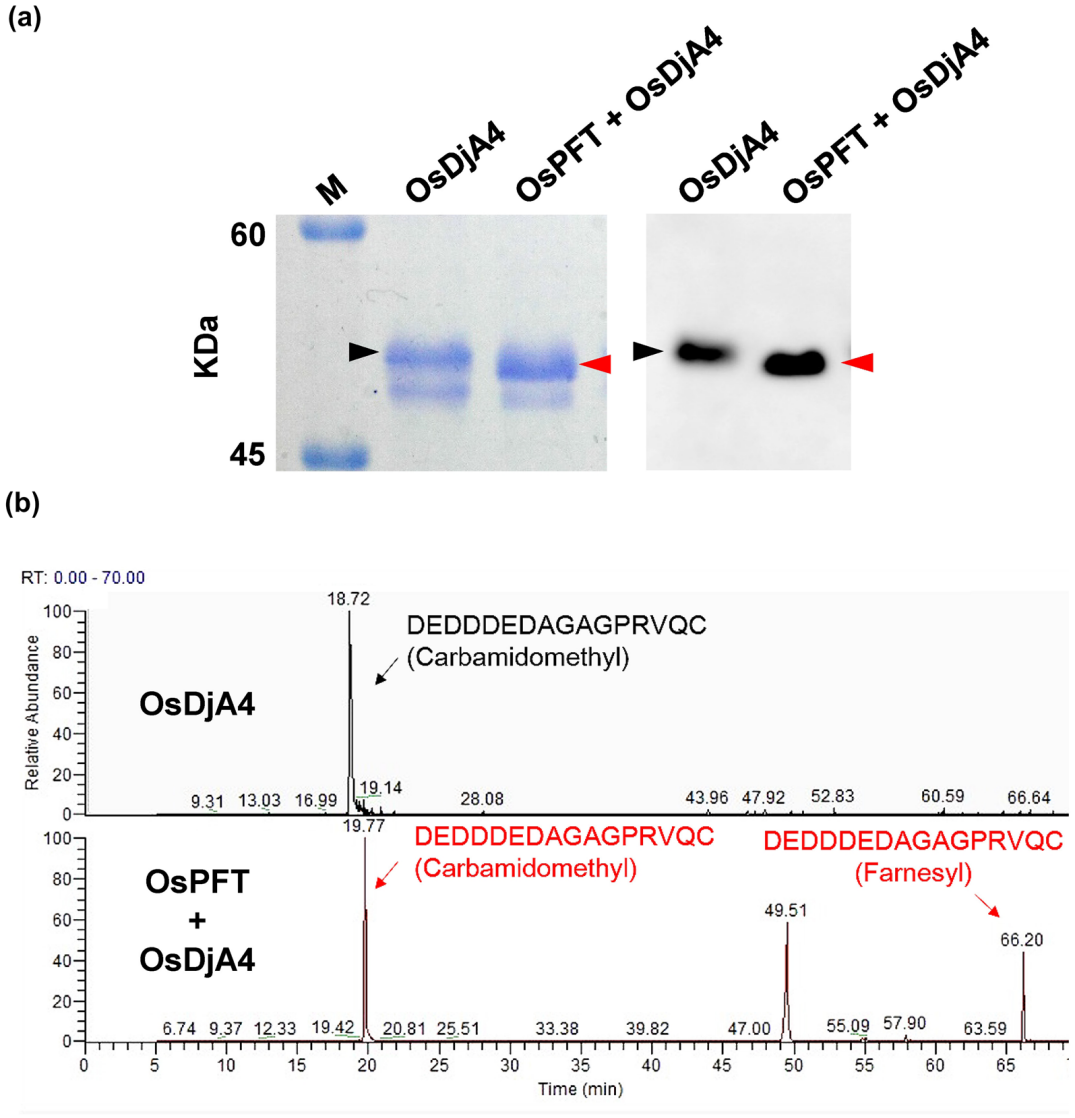
OsDjA4 was farnesylated in OsPFT-producing *E. coli* cells. **a** OsDjA4 produced in *E. coli* cells harboring either pGEX-OsDjA4 alone (OsDjA4) or both pGEX-OsDjA4 and pRSF-Duet1-OsPFT (OsDjA4 + OsPFT) was purified using Glutathione Sepharose 4B beads and run in duplicate, one for Coomassie blue staining and one for western blot detection, using SDS-PAGE. The OsDjA4 purified from pGEX-OsDjA4 and pRSF-Duet1-OsPFT co-transformed *E. coli* cells exhibited faster electrophoretic mobility than that from cells harboring pGEX-OsDjA4 alone, indicating OsDjA4 from co-transformed cells was farnesylated. **b** The extracted ion chromatograms (EIC) of the C-terminal peptide of OsDjA4 with and without farnesylation. The target peptide (DEDDDEDAGAGPRVQC) produced from the cells containing pGEX-OsDjA4 alone (upper panel, OsDjA4) showed only a non-farnesylated form with 874.8373 (2+) m/z at 18.72 min, but the target peptide produced from the cells containing pRSF-Duet1-OsPFT and pGEX-OsDjA4 (lower panel, OsPFT + OsDjA4) showed both non-farnesylated form with 874.8373 (2+) m/z at 19.77 min and farnesylated form with 948.4208 (2+) m/z at 66.20 min, respectively

After demonstrating the applicability of using *E. coli* to verify protein farnesylation in dicotyledonous plant species, we examined whether the same design was applicable to test protein farnesylation with components from monocotyledonous species. In this regard, cDNAs encoding rice PFTα (*OsPFTα*) and β (*OsPFTβ*) subunits were inserted into pRSF-Duet1 (pRSF-Duet1-OsPFT), and an AtJ3 homolog (*OsDjA4*) into pGEX-4T-1 (pGEX-OsDjA4). *OsPFTα* and *OsPFTβ* cDNAs were designed to contain a 6x His-tag sequence, whereas *OsDjA4* contains both a 6x His-tag and a GST sequence, and their expressions in *E. coli* were induced using IPTG (Fig. 1). Total proteins from *E. coli* cells co-transformed with pRSF-Duet1-OsPFT and pGEX-OsDjA4 with and without IPTG induction were extracted and separated using SDS-PAGE, followed by western blot analysis with an anti-His-tag antibody. The results indicated that OsPFTα, OsPFTβ, and OsDjA4 proteins were concomitantly produced in *E. coli* cells after IPTG induction (Fig. 4, Additional file 1: Fig. S4). The electrophoretic mobility of OsDjA4 in an SDS polyacrylamide gel from cells harboring pRSF-Duet1-OsPFT was faster than that from cells without pRSF-Duet1-OsPFT. Furthermore, MS analysis showed that the cysteine residue of the CaaX box in OsDjA4 isolated from cells containing pRSF-Duet1-OsPFT was attached to the farnesyl moiety, but not in cells lacking pRSF-Duet1-OsPFT (Fig. 5, Additional file 1: Fig. S5). Thus, PFTs from both monocotyledonous and dicotyledonous plants can maintain their molecular functions and their targets can be farnesylated in *E. coli*.

### Farnesylated AtJ3 produced in *E. coli* protects luciferase from heat inactivation

**Fig. 6:**
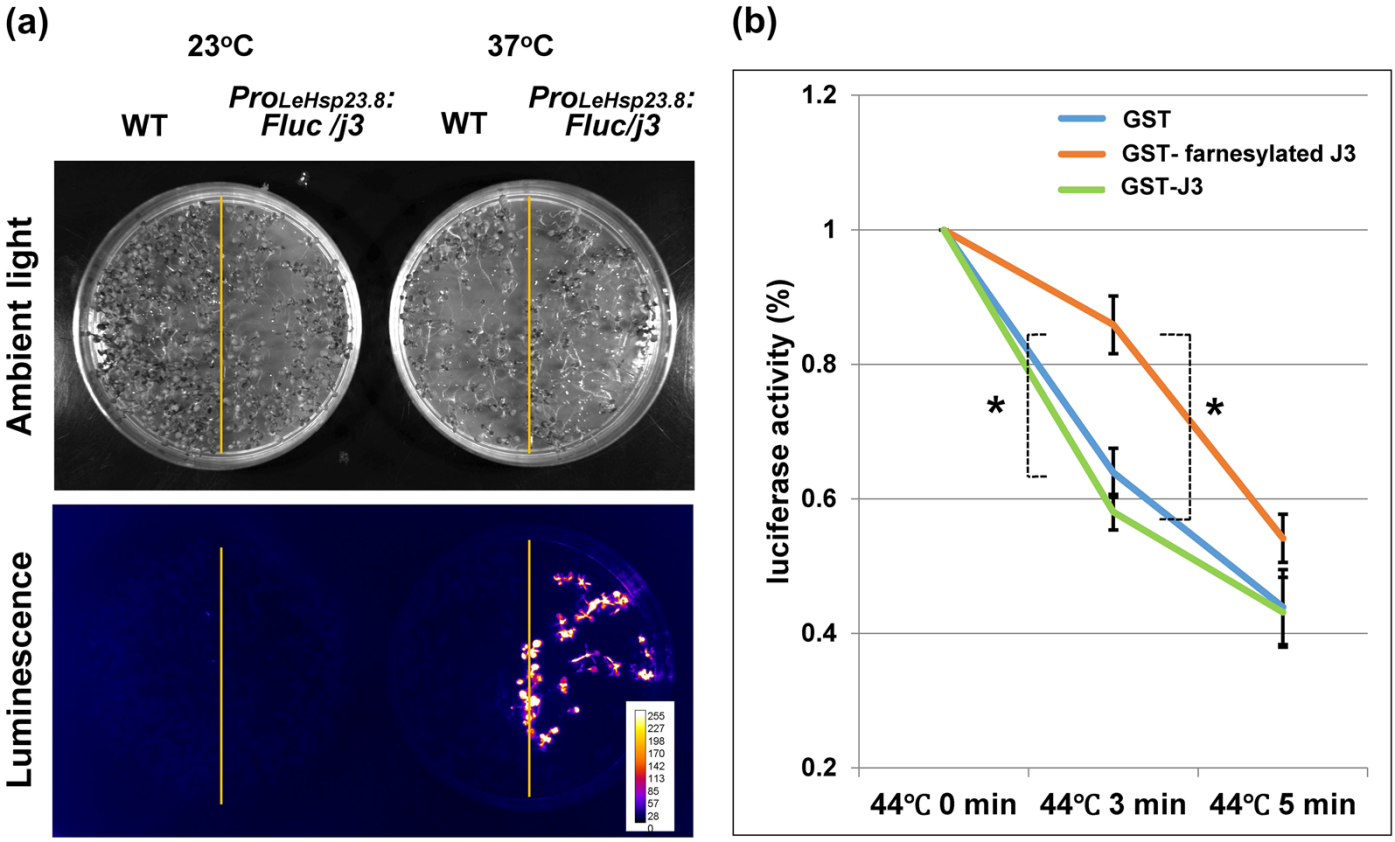
Farnesylated AtJ3 produced in *E. coli* prevents plant-synthesized luciferase from heat-induced denaturation. **a** The luminescence was observed in *Pro*_*LeHsp23.8*_-*Fluc/j3* after a 2 h incubation at 37 °C, indicating the induction of luciferase. **b** Cell lysate from 2 h heat-incubated *Pro*_*LeHsp23.8*_-*Fluc/j3* plants was mixed with proteins extracted from *E. coli* cells harboring both pGEX-AtJ3 and pRSF-Duet1-AtPFT (containing GST-tagged farnesylated AtJ3; GST-farnesylated J3), pGEX-AtJ3 alone (containing GST-tagged unfarnesylated AtJ3; GST-J3) and pGEX-4T-1 empty vector and pRSF-Duet1-AtPFT (containing GST). The mixtures were then incubated at 44 °C for different durations (0, 3, and 5 min), and the intensity of luciferase activities was monitored by measuring the luminescence. Results showed that after 3 min of heat stress, the mixture with farnesylated AtJ3 maintained a significantly higher luminescent signal (85.9%) than those with unfarnesylated AtJ3 (58.0%) or no AtJ3 (63.9%), indicating that the farnesylated AtJ3 produced in *E.coli* retained its ability to protect plant-synthesized proteins from thermal denaturation. Values represent the mean ± SD of three biological replicates. *P ≤ 0.01 (unpaired t-test)

A recent study showed that Arabidopsis with unfarnesylated AtJ3 accumulated more heat-induced insoluble proteins than Arabidopsis with farnesylated AtJ3, indicating that farnesylation is critical for AtJ3 to exert its co-chaperone function to protect proteins from heat denaturation [35]. To examine whether the farnesylated AtJ3 produced in *E. coli* maintains this function, we generated a transgenic Arabidopsis line in an AtJ3-null mutant background with cDNA encoding thermolabile firefly luciferase (Fluc), whose expression was under the control of the heat-inducible promoter *Pro*_*LeHsp23.8*_ [36]. Luminescence was detected after the transgenic plants were incubated at 38 °C for 2 h (Fig. 6a). Cell lysates from heat-treated transgenic plants were prepared and mixed with either farnesylated or unfarnesylated AtJ3 produced in and isolated from *E. coli* for a luciferase-based protein denaturation assay. The mixtures were incubated at 44 °C for various durations, and the luminescence intensity of the mixtures was monitored. The results showed that the luminescence intensity of the lysate mixed with farnesylated AtJ3 decayed slower than that with unfarnesylated AtJ3, whereas the luminescence intensity of the lysate mixed with unfarnesylated AtJ3 was similar to that of the lysate (Fig. 6b). These results indicate that farnesylated AtJ3 produced in *E. coli* maintained its native molecular function, which was able to protect proteins from heat-induced damage, and strengthened the notion that farnesylation plays a critical role in AtJ3 to exert its protein-protecting function.

### Farnesylated AtJ3 enhances thermotolerance in *E. coli*

**Fig. 7:**
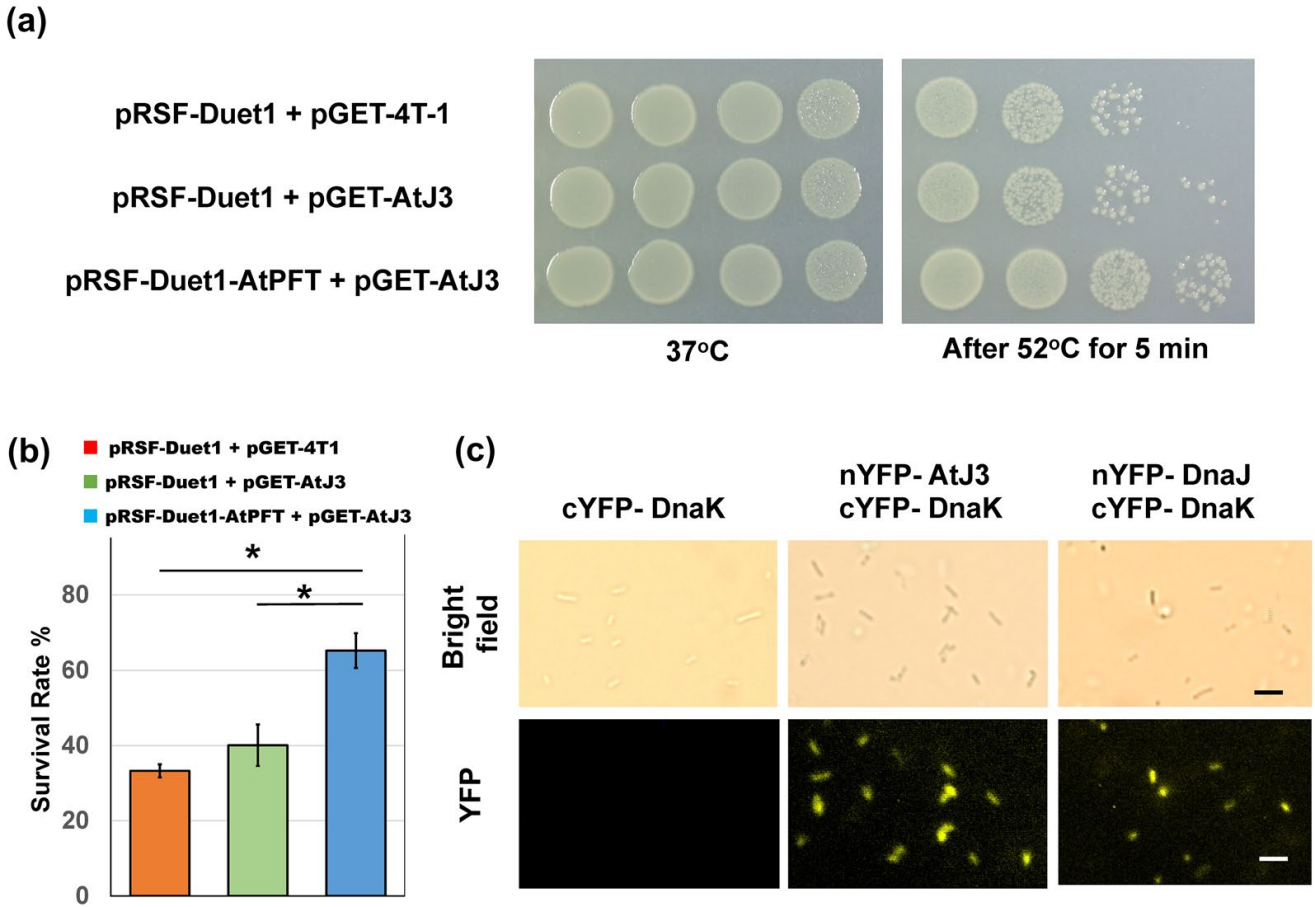
Enhancement of thermotolerance in *E. coli* as a result of the presence of farnesylated AtJ3. **a** *E.coli* strains harboring pGEX-4T-1 and pRSF-Duet1 empty vectors (contains no AtJ3), pGEX-AtJ3 and pRSF-Duet1 empty vector (contains unfarnesylated AtJ3), and both pGEX-AtJ3 and pRSF-Duet1-AtPFT (contains farnesylated AtJ3) were induced with IPTG then heat stressed at 52 °C for 5 min. After the treatment, ten-fold dilutions of *E. coli* cells were spotted on LB plates and incubated at 37 °C for 16 h before pictures were taken. **b** The survival rate of *E. coli* cells containing farnesylated AtJ3 was 65.2% compared to 39.8% for cells containing unfarnesylated AtJ3 and 33.7% of those containing no AtJ3. Values represent the mean ± SD of three biological replicates. *P ≤ 0.01 (unpaired t-test). **c** *E. coli* cells either co-expressed with nYFP-DnaJ and cYFP-DnaK or nYFT-AtJ3 and cYFP-DnaK exhibited BiFC fluorescence, indicating AtJ3 can interact with DnaK in *E. coli*. Bar = 3 μm

The farnesylation of Arabidopsis HSP40 (AtJ3) plays a critical role in plant heat tolerance [35]. Although *E. coli* cells produce HSP40 (DnaJ), but not PFT, it is tempting to test whether HSP40 farnesylation can enhance thermotolerance in *E. coli*. *E. coli* cells containing both pRSF-Duet1-AtPFT and pGEX-AtJ3, pRSF-Duet1 and pGEX-AtJ3, and pRSF-Duet1 and pGEX-4T-1 were induced with IPTG then subjected to 52 °C heat stress for 5 min, and their survival rates were calculated. The results showed that the survival rate of cells containing both pRSF-Duet1-AtPFT and pGEX-AtJ3 was significantly higher (65.2%) than those containing the pRSF-Duet1 empty vector and pGEX-AtJ3 (39.8%) or pRSF-Duet1 and pGEX-4T-1 empty vectors (33.7%), demonstrating that farnesylated AtJ3 can enhance thermotolerance in *E. coli*, as similar to Arabidopsis (Fig. 7a, b). As a co-chaperone, HSP40 functions together with the major molecular chaperone HSP70. It has been demonstrated that AtJ3 interacts with Arabidopsis AtHSP70-4 [35]. To better elucidate the mechanism underlying the enhanced thermotolerance of *E. coli* conferred by farnesylated AtJ3, we tested whether AtJ3 interacted with *E. coli* HSP70 (DnaK). A construct expressing both nYFP-tagged AtJ3 (nYFP-AtJ3) and cYFP-tagged DnaK (cYFP-DnaK) was constructed and transformed into *E. coli*. The interaction between AtJ3 and DnaK was examined by using a bimolecular fluorescence complementation (BiFC) assay. The results showed that cells co-expressing nYFP-AtJ3 and cYFP-DnaK generated BiFC fluorescence, indicating that AtJ3 interacted with DnaK in *E. coli*. (Fig. 7c).

## Discussion

Protein farnesylation, catalyzed by PFT, is a post-translational modification in which a 15-carbon farnesyl lipid is added to a protein. In plants, this process is crucial for the proper functioning of many proteins, including those involved in the regulation of plant development [16, 18, 40, 41], response to environmental stresses [11, 42], and defense against pathogens [19]. Understanding its mechanisms and consequences is important for developing new strategies to improve crop yield and plant resistance to pathogens. It is necessary to identify the farnesylated proteins to achieve this goal. Although the potential target of PFT can be predicted by the presence of a PFT-recognizing CaaX box at the C-terminus of the protein, methods traditionally used to verify the farnesylation of a specific protein are cumbersome. As PFT is not present and protein farnesylation does not occur in prokaryotic cells, this study aimed to develop an efficient *E. coli* system for characterizing farnesylated plant proteins. Farnesylated plant proteins are generally subjected to post-prenylation processing, that is, removal of the three terminal amino acids and addition of a methyl group. *E. coli* lacks this machinery and farnesylated AtJ3 appears to be functional in protecting both plant and *E. coli* cells suggesting that post-prenylation modification is not crucial for AtJ3 chaperone function. In addition to AtJ3, we also successfully used the *E. coli* farnesylation system to produce and purify farnesylated Arabidopsis AtFP3 (At5g63530; Additional file 1: Fig. S3). This protein is known to bind transition metals and is involved in plant responses to heavy metals [43, 44]. These results indicate that the *E. coli*-based plant protein farnesylation system can be used to verify PFT targets and to produce farnesylated plant proteins with their native molecular function for various biochemical, molecular, and physiological studies.

*E. coli* has a single HSP40 (DnaJ) that participates actively in the heat stress response by preventing the aggregation of heat-denatured proteins or by disaggregating proteins. Because *E. coli* has no PFT and its DnaJ lacks a CaaX motif, this protective function does not require DnaJ farnesylation. Nevertheless, in the present study, the presence of farnesylated AtJ3 enhanced the thermotolerance of *E. coli*. The major role played by HSP40 is as a co-chaperone of HSP70. It can initiate contact with heat-denatured proteins and bring them to HSP70[38]. Only when HSP40 and its target are present simultaneously can the ATPase activity of HSP70 be fully stimulated to convert HSP70-bond ATP to ADP, which is required for HSP70 to refold denatured proteins [45]. From this viewpoint, farnesylated AtJ3, which has higher hydrophobicity, may interact better with the exposed hydrophobic stretches of heat-denatured proteins and transport the substrates to *E. coli* HSP70 (DnaK). BiFC experiments showed that AtJ3 interacted with DnaK, supporting this possibility. Alternatively, HSP40 suppresses protein aggregation in an autonomous and DnaK-independent fashion [39,40]. Hence, the presence of farnesylated AtJ3 alone may enhance thermotolerance in *E. coli*. In either scenario, this is consistent with the notion that farnesylation promotes protein-protein interactions, which is a major characteristic of HSP40.

Protein farnesylation is one of the two types of protein prenylation. The second type is protein geranylgeranylation, which is catalyzed by protein geranylgeranyl transferase (PGGT). Both PFT and PGGT recognize the CaaX motif and attach lipids to cysteine residues. Nevertheless, PFT and PGGT each have their own preferred “aaX” amino acid composition [46–48]. Theoretically, the plant protein farnesylation system in *E. coli* demonstrated in this study can be used to construct plant protein geranylgeranylation systems by simply substituting PGGT with PFT. However, *E. coli* cells readily contain farnesyl pyrophosphate for PFT but not geranylgeranyl pyrophosphate for PGGT. More genetic modifications are required, yet it is foreseeable that geranylgeranylation to realize plant protein geranylgeranylation in *E. coli*. After that, a comprehensive study of plant protein prenylation, including the mechanisms for preferential recognition of “aaX” of PFT and PGGT and the different effects of a protein being farnesylated or geranylgeranylated, can be conducted in *E. coli*.

## Conclusions

This study demonstrated the applicability of producing functional plant PFT and executing plant protein farnesylation in *E. coli* and that the farnesylated plant protein maintained its native function. This greatly facilitates verifying and characterizing PFT targets and elucidates the significance and network of farnesylation-mediated regulation of plant growth, development, and responses to biotic and abiotic stresses.

### Supplementary Information

**Additional file 1: Table S1.** The primers used in this study. **Fig S1.** Co-expression of AtPFTα, AtPFTβ, and AtJ3 in *E. coli*. **Fig S2.** Characterization of farnesylated AtJ3 produced in and purified from *E.coli*. **Fig S3.** Characterization of farnesylated ATFP3 produced in and purified from *E.coli*. **Fig S4.** Co-expression of OsPFTα, OsPFTβ, and OsDjA4 in *E. coli*. **Fig S5.** Characterization of farnesylated OsDjA4 produced in and purified from *E.coli*.

## Data Availability

The materials will be available on request.
