# Supplementary material for: A plant protein farnesylation system in prokaryotic cells reveals Arabidopsis AtJ3 produced and farnesylated in E. coli maintains its function of protecting proteins from heat inactivation
Source: Plant Methods. 2023 Oct 26;19:113. doi: 10.1186/s13007-023-01087-x (PMC10604809; doi:10.1186/s13007-023-01087-x)
Supplement: Supplementary file 1 — Additional file 1: Table S1. The primers used in this study. Fig S1. Co-expression of AtPFTα, AtPFTβ, and AtJ3 in E. coli. Fig S2. Characterization of farnesylated AtJ3 produced in and purified from E.coli. Fig S3. Characterization of farnesylated ATFP3 produced in and purified from E.coli. Fig S4. Co-expression of OsPFTα, OsPFTβ, and OsDjA4 in E. coli. Fig S5. Characterization of farnesylated OsDjA4 produced in and purified from E.coli. [file 13007_2023_1087_MOESM1_ESM.pdf]

**Additional file**

# A plant protein farnesylation system in prokaryotic cells reveals Arabidopsis AtJ3 produced and farnesylated in *E. coli* maintains its function of protecting proteins from heat inactivation.

Jia-Rong Wu†, Rida Zohra†, Thi Ngoc Kieu Duong, Ching-Hui Yeh, Chung-An Lu*, Shaw-Jye Wu*

*Corresponding Author:

Dr. Shaw-Jye Wu
Department of Life Sciences,
National Central University
300 Jhong-Da Road, Jhong-Li District, Taoyuan City 32001, Taiwan
Tel.: +886-3-4227971, Fax: +886-3-4228482
Email: jyewu@cc.ncu.edu.tw

Dr. Chun-An Lu
Department of Life Sciences,
National Central University
300 Jhong-Da Road, Jhong-Li District, Taoyuan City 32001, Taiwan
Tel.: +886-3-4227151 ext. 65067, Fax: +886-3-4228482
Email: chungan@cc.ncu.edu.tw

**This file includes:**

Table S1. The primers used in this study.
Fig. S1. Co-expression of AtPFTα, AtPFTβ, and AtJ3 in *E. coli*.
Fig. S2. Characterization of farnesylated AtJ3 produced in and purified from *E.coli.*
Fig. S3. Characterization of farnesylated ATFP3 produced in and purified from *E.coli.*
Fig. S4. Co-expression of OsPFTα, OsPFTβ, and OsDjA4 in *E. coli*.
Fig. S5. Characterization of farnesylated OsDjA4 produced in and purified from *E.coli.*

**Table S1**: Primers used in this study. Restriction enzyme sites are underlined.

| **Name** | **Sequence** |
|---|---|
| AtPFT-α-BamHI-F | CGGATCCGATGAATTTCGACGAGACCGTGC |
| AtPFT-α-PstI-R | GCTGCAGTCAAATTGCTGCCACTGTAATC |
| AtPFT-β-6xHis-KpnI-F | GGGGTACCCATCACCATCACCATCACATGCCAGTAGTAACCCGCTTGATTC |
| AtPFT-β-PacI-R | CCTTAATTAATCATGCTGCTTTAAAGAAGAACTCG |
| AtJ3-6xHis-BamHI-F | CGGGATCCCATCACCATCACCATCACATGTTCGGTAGAGGACCCTCG |
| AtJ3-stop-XhoI-R | CCGCTCGAGTTACTGCTGGGCACATTGCACCC |
| OsPFT-α-BamHI-F | CGGGATCCGATGGCGCCGTCGTCGACGTCGT |
| OsPFTα-EcoRI-R | GGAATTCTCAGGTTTGAGAAGAAATAGTGG |
| OsPFTβ-6xHis-KpnI-F | GGGGTACCCATCACCATCACCATCACATGGTTCGCCGCCTTTCGGTGA |
| OsPFT-β-XhoI-R | CCGCTCGAGCTAGCTTGAAAAGAACTCATAGG |
| OsDjA4-6xHis-SmaI-F | TTCCCCGGGTCATCACCATCACCATCACATGTACGGACGCATGCCAAAGAAG |
| OsDjA4-stop-NotI-R | ATTTGCGGCCGCTTACTGCTGGGCACACTGTA |

| | |
|---|---|
| DnaJ-attB3 | GGGGACAACTTTGTATAATAAAGTTGGAATGGCTAAGCAAGATTATTACG |
| DnaJ-stop-attB2 | GGGGACCACTTTGTACAAGAAAGCTGGGTTTAGCGGGTCAGGTCGTCAAA |
| DnaK-attB1 | GGGGACAAGTTTGTACAAAAAAGCAGGCTTAATGGGTAAAATAATTGGTATCG |
| DnaK-attB4 | GGGGACAACTTTGTATAGAAAAGTTGGGTTTATTTTTTGTCTTTGACTTCTTC |
| AtJ3-attB3 | GGGGACAACTTTGTATAATAAAGTTGGAATGTTCGGTAGAGGACCCTCGAA |
| AtJ3-stop-attB2 | GGGGACCACTTTGTACAAGAAAGCTGGGTTTACTGCTGGGCACATTGCACCC |
| pBiFCt-2in1-NN-(531-551)-BamHI-F | CGGGATCCGATGGTGAGCAAGGGCGAGGAG |
| AtJ3-stop-EcoRI-R | GGAATTCTTACTGCTGGGCACATTGCACCC |
| DnaJ-stop-EcoRI-R | GGAATTCTTAGCGGGTCAGGTCGTCAAA |
| pBiFCt-2in1-NN-(3906-3926)-NdeI-F | GGAATTCCATATGATGGACAAGCAGAAGAACGGC |
| DnaK-stop-XhoI-R | CCGCTCGAGTTATTTTTTGTCTTTGACTTCTTC |
| AtFP3-6xHis-BamHI-F | CGGGATCCCATCACCATCACCATCACATGGGAGAGGAAGAGAAGAAACC |
| AtFP3-stop-EcoRI-R | GGAATTCTTACATTACAGTACATGCATTTGG |

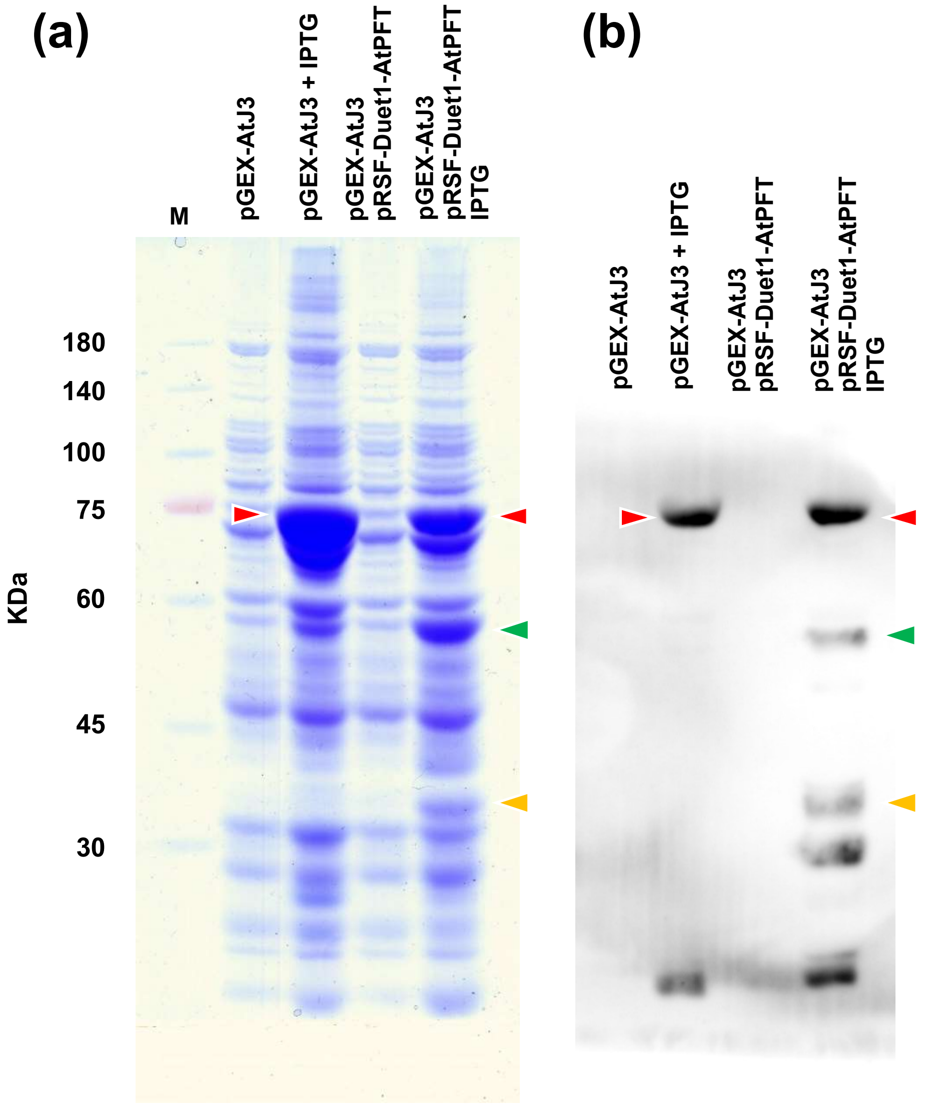


**Fig. S1.** Co-expression of AtPFTα, AtPFTβ, and AtJ3 in *E. coli*. (a) Coomassie blue staining of a gel with proteins extracted from *E. coli* cells containing either pGEX-At3J alone or both pGEX-At3J and pRSF-Duet1-AtPFT before or after IPTG induction. (b) The protein samples in (a) were analyzed using western blot analysis with anti-6xHis-tag antibody. The molecular size of GST-6xHis-AtJ3 (72.67 kDa, red arrowhead), 6xHis-AtPFTβ (54.99 kDa, green arrowhead), and 6xHis-AtPFTα (38.78 kDa, orange arrowhead) are indicated.

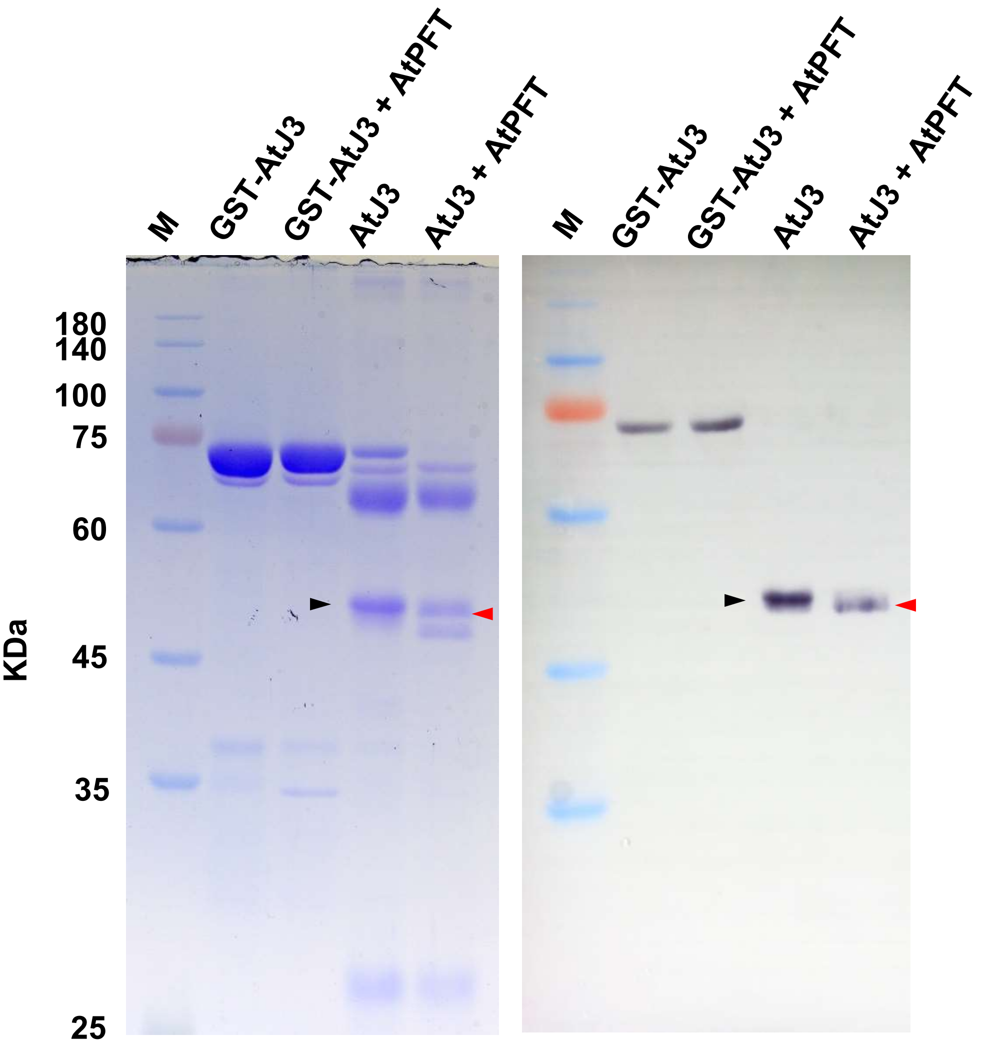


**Fig. S2.** Characterization of farnesylated AtJ3 produced in and purified from *E.coli*. GST-tagged AtJ3 was isolated from *E. coli* cells expressing either pGEX-AtJ3 alone or both pGEX-AtJ3 and pRSF-Duet1-AtPFT using Glutathione Sepharose 4 B beads. The purified AtJ3 was also digested with thrombin to observe the clear mobility shift. The AtJ3 with and without GST tag was run in duplicate, one for Coomassie blue staining (left panel) and one for western blot detection (right panel), using SDS-PAGE. The AtJ3 protein purified from *E. coli* cells co-transformed with pGEX-AtJ3 and pRSF-Duet1-AtPFT (AtPFT + AtJ3, indicated by the red arrowhead) exhibited a faster electrophoretic mobility compared to the AtJ3 protein obtained from cells harboring pGEX-AtJ3 alone (AtJ3, indicated by the black arrowhead), indicating AtJ3 from co-transformed cells was farnesylated.

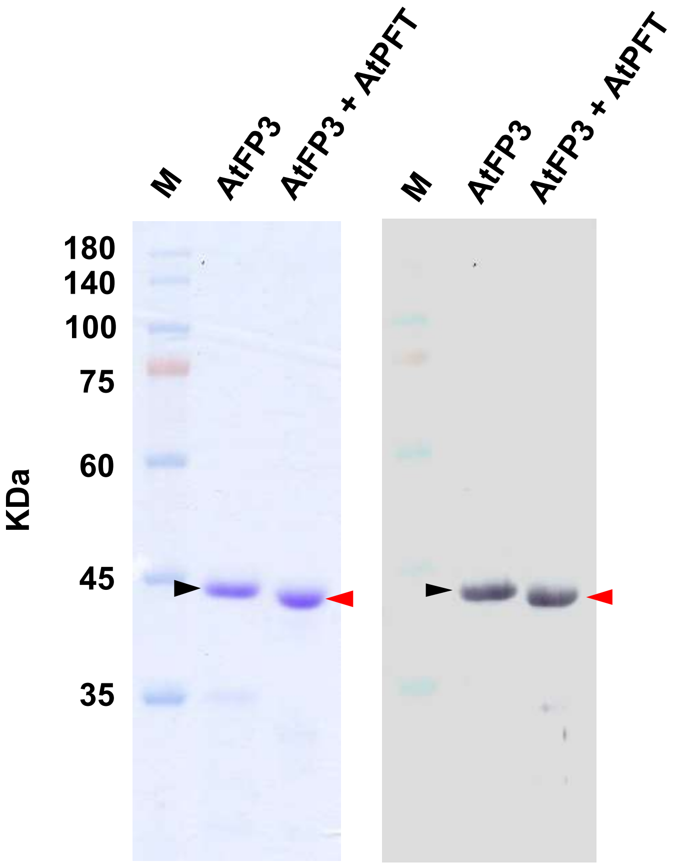


**Fig. S3.** Characterization of farnesylated AtFP3 produced in and purified from *E.coli*. GST-tagged AtFP3 was isolated from *E. coli* cells expressing either pGEX-AtFP3 alone or both pGEX-AtFP3 and pRSF-Duet1-AtPFT using Glutathione Sepharose 4 B beads. Using SDS-PAGE, the isolated AtFP3 was digested with thrombin and ran two times, once for Coomassie blue staining and another time for western blot detection. The electrophoretic mobility of AtFP3 purified from *E. coli* cells co-transformed with pGEX-AtFP3 and pRSF-Duet1-AtPFT (AtFP3+AtPFT, red arrowhead) was faster compared to cells containing only pGEX-AtFP3 (AtFP3, indicated by the black arrowhead), indicating AtFP3 from co-transformed cells was farnesylated.

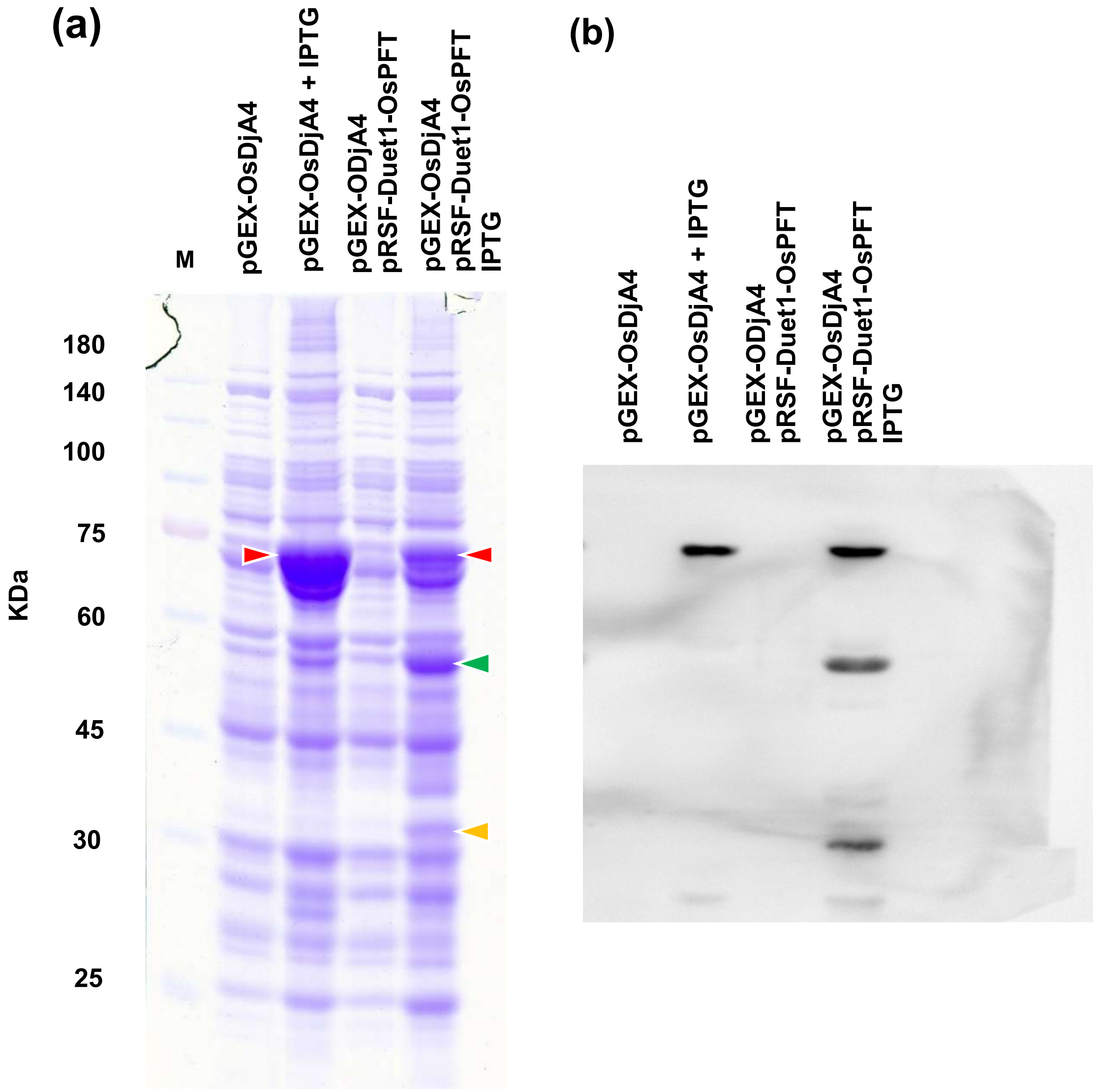


**Fig. S4.** Co-expression of OsPFTα, OsPFTβ, and OsDjA4 in *E. coli*. (a) Coomassie blue staining of a gel with proteins extracted from *E. coli* cells harboring either pGEX-OsDjA4 alone or both pGEX-OsDjA4 and pRSF-Duet1-OsPFT, before or after IPTG induction. (b) The protein samples in (a) were analyzed using western blot analysis with an anti-6xHis-tag antibody. The molecular size of GST-6xHis-OsDjA4 (73.11 kDa, red arrowhead), 6xHis-OsPFTβ (53.43 kDa, green arrowhead), and 6xHis-AtPFTα (38.2 kDa, orange arrowhead) are indicated.

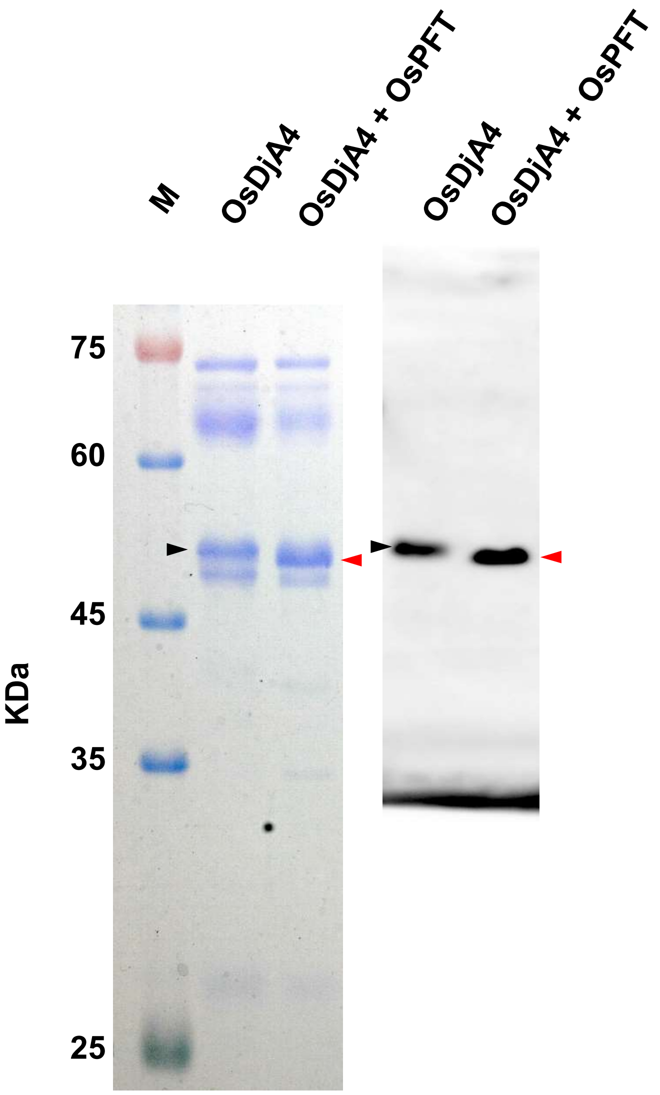


**Fig. S5.** Characterization of farnesylated OsDjA4 produced in and purified from *E.coli*. GST-tagged OsDjA4 was isolated from *E. coli* cells expressing either pGEX- OsDjA4 alone or both pGEX- OsDjA4 and pRSF-Duet1-OsPFT using Glutathione Sepharose 4 B beads. The purified OsDjA4 was digested with thrombin and run in duplicate, one for Coomassie blue staining (left panel) and one for western blot detection (right panel), using SDS-PAGE. The electrophoretic mobility of OsDjA4 purified from *E. coli* cells co-transformed with pGEX-OsDjA4 and pRSF-Duet1-OsPFT (OsDjA4+OsPFT, red arrowhead) was faster compared to cells containing only pGEX-OsDjA4 (OsDjA4, indicated by the black arrowhead), indicating OsDjA4 from co-transformed cells was farnesylated.
